# RUNX1/ETO blocks selectin-mediated adhesion via epigenetic silencing of PSGL-1

**DOI:** 10.1038/oncsis.2015.6

**Published:** 2015-04-13

**Authors:** K Ponnusamy, N Kohrs, A Ptasinska, S A Assi, T Herold, W Hiddemann, J Lausen, C Bonifer, R Henschler, C Wichmann

**Affiliations:** 1 Department of Transfusion Medicine, Cell Therapeutics and Hemostaseology, Ludwig-Maximilian University Hospital, Munich, Germany; 2 Institute of Transfusion Medicine and Immunohematology, Goethe University, Frankfurt, Germany; 3 Institute for Tumor Biology and Experimental Therapy, Georg-Speyer-Haus, Frankfurt, Germany; 4 School of Cancer Sciences, University of Birmingham, Birmingham, UK; 5 Department of Internal Medicine 3, Ludwig-Maximilian University Hospital, Munich, Germany

## Abstract

RUNX1/ETO (RE), the t(8;21)-derived leukemic transcription factor associated with acute myeloid leukemia (AML) development, deregulates genes involved in differentiation, self-renewal and proliferation. In addition, these cells show differences in cellular adhesion behavior whose molecular basis is not well understood. Here, we demonstrate that RE epigenetically silences the gene encoding P-Selectin Glycoprotein Ligand-1 (PSGL-1) and downregulates PSGL-1 expression in human CD34+ and murine lin− hematopoietic progenitor cells. Levels of PSGL-1 inversely and dose-dependently correlate with RE oncogene levels. However, a DNA-binding defective mutant fails to downregulate PSGL-1. We show by ChIP experiments that the *PSGL-1* promoter is a direct target of RE and binding is accompanied by high levels of the repressive chromatin mark histone H3K27me3. In t(8;21)+ Kasumi-1 cells, PSGL-1 expression is completely restored at both the mRNA and cell surface protein levels following RE downregulation with short hairpin RNA (shRNA) or RE inhibition with tetramerization-blocking peptides, and at the promoter H3K27me3 is replaced by the activating chromatin mark H3K9ac as well as by RNA polymerase II. Upregulation of PSGL-1 restores the binding of cells to P- and E-selectin and re-establishes myeloid-specific cellular adhesion while it fails to bind to lymphocyte-specific L-selectin. Overall, our data suggest that the RE oncoprotein epigenetically represses *PSGL-1* via binding to its promoter region and thus affects the adhesive behavior of t(8;21)+ AML cells.

## Introduction

RUNX1/ETO (RE), the t(8;21)-derived fusion protein, is present in 12% of *de novo* acute myeloid leukemia (AML) cases and up to 40% of M2 subtype AMLs according to the French–American–British classification.^1^ Recently, a truncated form of RE (REtr), which lacks the C-terminal N-CoR/SMRT-interacting domain, has been identified, recapitulating a naturally occurring highly leukemogenic splice variant RE9a observed in AML patients.^2, 3^ RE harbors the DNA-binding domain of RUNX1 fused to the nearly entire nuclear co-repressor ETO protein. RE heterodimerizes with CBFβ for efficient binding to DNA in complex with other transcription regulators that causes deregulation of normal myelopoiesis.^1, 4^ The ETO region acts as a dominant repressor of RUNX1 target genes by recruiting nuclear co-repressors such as N-CoR, SMRT, mSIN3A and histone deacetylases.^5, 6, 7, 8^ RE directly represses several targets such as PU.1, CEBPα, microRNA-223, granulocyte–macrophage colony-stimulating factor and neutrophil elastase.^9, 10, 11, 12^ However, RE can also activate the transcription of certain genes such as *p21/WAF/Cip1, ID1* and *EGR1* via recruitment of p300.^12^ Likewise, RE epigenetically deregulates genes involved in proliferation, self-renewal and differentiation of hematopoietic stem and progenitor cells, which results in a differentiation block at the myeloblastic stage.^13^

Adhesion molecules have a pivotal role in hematopoietic stem and progenitor cell trafficking and steady-state hematopoiesis.^14^ The expressions of hematopoietic stem and progenitor cell homing receptors such as VLA-4, LFA-1, P-Selectin Glycoprotein Ligand-1 (PSGL-1) and CD44 are frequently deregulated in malignant hematopoietic cells.^15, 16, 17, 18^ Moreover, expressions of CD44, VLA-4 and LFA-1 have been found to be directly regulated by RE.^15, 17, 19^ The transmembrane protein PSGL-1 is a 220-kDa disulfide-linked homodimeric sialomucin expressed on the surface of activated endothelial cells, myeloid cells and lymphoid cells.^20^ It is the principal ligand for P (platelet)-selectin; however, under physiological flow conditions, it also binds to E (endothelial)- and L (leukocyte) selectin.^21, 22^ Selectin recognition is critically dependent on post-translational modifications such as sialylation, fucosylation and O-glycosylation of PSGL-1.^20, 23, 24^ PSGL-1 also harbors binding sites for the chemokines CCL19 and CCL21 and efficiently regulates the homing of T cells to secondary lymphoid organs.^25, 26^ The PSGL-1/P-selectin interaction contributes to the rolling of hematopoietic cells on endothelial cells followed by migration into tissues, thereby regulating immunity and steady-state hematopoiesis.^21, 27, 28^ PSGL-1-deficient leukocytes show impaired tethering to and rolling on P- and E-selectin *in vivo.*^29^ Furthermore, blocking the N-terminal region of PSGL-1 with monoclonal antibodies abolishes leukocyte rolling on P- and L-selectin.^30^

In this report, we show that RE directly interacts with the *PSGL-1* promoter region and epigenetically represses *PSGL-1* in hematopoietic progenitor cells. The result of this repression is an impediment of cellular adhesion, which is completely restored after depletion of RE. This demonstrates that the deregulation of *PSGL-1* expression and other adhesion molecules is an important feature of t(8;21)+ AML.

## Results

### PSGL-1 is absent in t(8;21)+ AML cells

**Figure 1:**
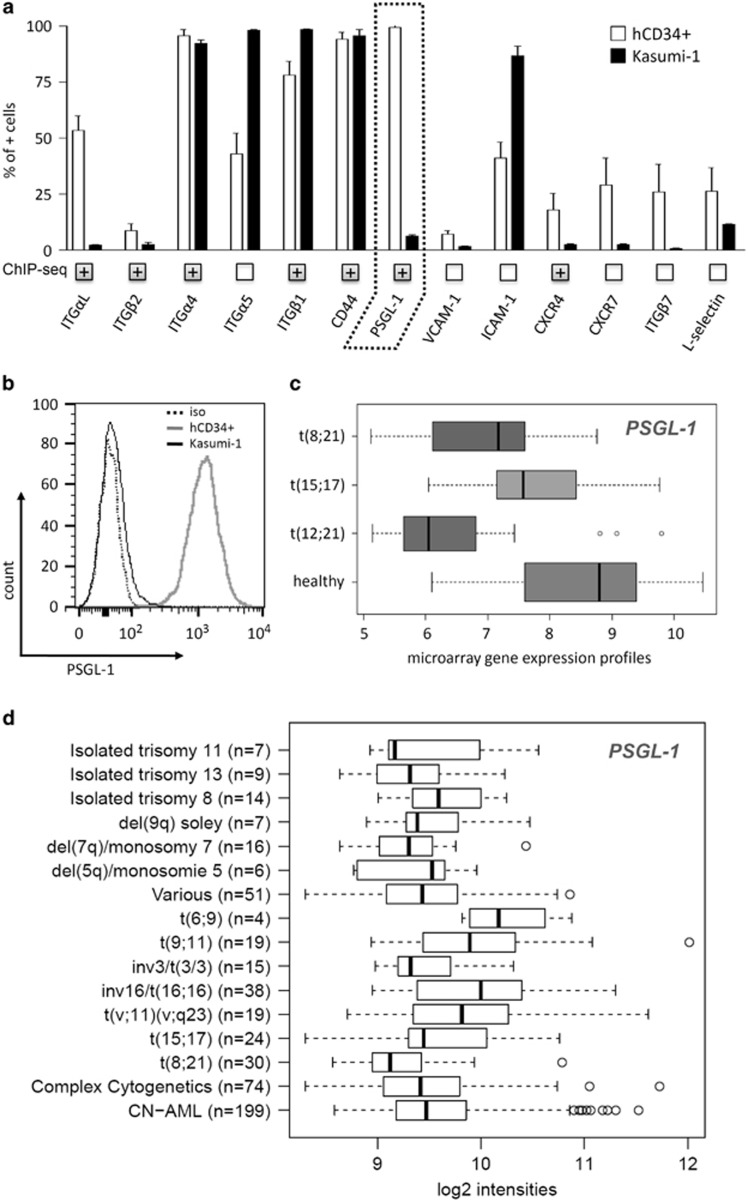
Expression levels of PSGL-1 in t(8;21)+ leukemic cells. (**a**) Cell surface expression patterns of adhesion molecules in Kasumi-1 cells and human hematopoietic CD34+ cells as assessed via flow cytometry (*n*=3). (**b**) Histogram of cell surface expression levels of PSGL-1 in t(8;21)+ Kasumi-1 cells and healthy donor-derived human CD34+ cells as analyzed via flow cytometry. (**c**) Expression levels of *PSGL-1* mRNA in patient-derived primary leukemic cells based on microarray data analysis.^32^ (**d**) *PSGL-1* mRNA expression in patient-derived primary leukemic cells categorized by karyotypes. Data are shown as the mean±s.e.m.

To identify adhesion molecules regulated by RE, we analyzed the adhesion molecule expression pattern of Kasumi-1 cells compared with human CD34+ cells obtained from peripheral blood apheresis of healthy donors (Figure 1a). The integrin *LFA-1* (*CD11a*, *ITGαL*) has been reported as a directly repressed RE target gene^15^ and was found to be expressed at low levels in Kasumi-1 cells (Figure 1a). Differences were also observed for ITGα5, ITGß1, ICAM-1, CXCR4, CXCR7, ITGß7 and L-selectin. Most strikingly, Kasumi-1 cells express barely detectable levels of the sialomucin PSGL-1 (Figures 1a and b). PSGL-1 was also identified as a potential RE target via genome-wide chromatin immunoprecipitation (ChIP)-sequencing experiments with Kasumi-1 cells and t(8;21)+ patient material.^31^ In agreement with our observations, a publically available gene array database has revealed low *PSGL-1* expression levels in t(8;21)+ primary leukemic cells compared with healthy counterparts (Figure 1c).^32^ A further analysis of a large AML gene array data set categorized by karyotypes also revealed low PSGL-1 expression in t(8;21)+ samples (Figure 1d).

### RE downregulates PSGL-1 expression in hematopoietic progenitor cells

**Figure 2:**
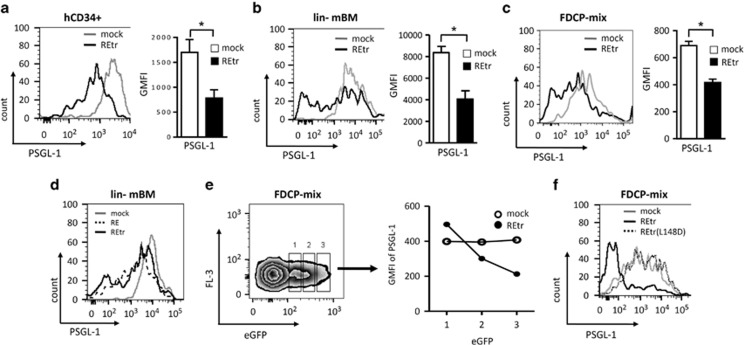
Regulation of PSGL-1 expression in enriched hematopoietic progenitor cells. Expression of PSGL-1 in (**a**) human primary hematopoietic CD34+ progenitor cells, (**b**) lin− mBM cells and (**c**) FDCP-mix cells as analyzed via flow cytometry on day 4 post transduction. (**d**) PSGL-1 levels of RE- and REtr-expressing lin− mBM cells on day 4 post transduction. (**e**) After transduction different levels of eGFP expression in FDCP-mix cells were gated as population 1, 2 and 3. PSGL-1 expression levels were measured for the corresponding gated cells. The data show representative results obtained out of three experiments. (**f**) A representative histogram of PSGL-1 expression in a DNA-binding defective mutant form of REtr(L148D)-transduced cells. *P<0.05. *n*=3.

To understand whether RE directly regulates PSGL-1, we overexpressed REtr in various hematopoietic progenitor cells via lentiviral vector transduction. On day 4 after transduction, PSGL-1 expression levels in hematopoietic progenitor cells were analyzed using flow cytometry. REtr downregulated PSGL-1 cell surface expression in mobilized human hematopoietic CD34+ progenitor cells compared with mock-transduced control cells (Figure 2a). REtr also downregulated PSGL-1 in murine lineage-negative bone marrow-derived primary hematopoietic progenitor (lin− mBM) cells and in a factor-dependent multipotent FDCP-mix progenitor cell line (Figures 2b and c). Likewise, full-length RE repressed PSGL-1 expression in lin− mBM cells (Figure 2d). Together, these data indicate that RE is a potent repressor of PSGL-1 expression in hematopoietic progenitor cells. We co-expressed enhanced green fluorescent protein (eGFP) from the same construct and gated cells expressing eGFP at low, medium and high levels to elucidate the dosage dependency of REtr on PSGL-1 regulation. Compared with empty vector-transduced cells, the expression levels of REtr inversely correlated with PSGL-1 levels (Figure 2e). Likewise, dose-dependent downregulation of PSGL-1 by REtr was also observed in primary human CD34+ progenitor cells (Supplementary Figure 1). Next, we examined whether RE required its DNA-binding function to regulate PSGL-1 expression. The DNA-binding-defective mutant REtr(L148D)^33, 34^ completely failed to regulate PSGL-1 in FDCP-mix cells (Figure 2f). Overall, we found that RE downregulates PSGL-1 expression in hematopoietic progenitor cells in a DNA-binding- and dose-dependent manner.

### Depletion and inhibition of RE induce complete re-expression of PSGL-1 in t(8;21)+ Kasumi-1 cells

**Figure 3:**
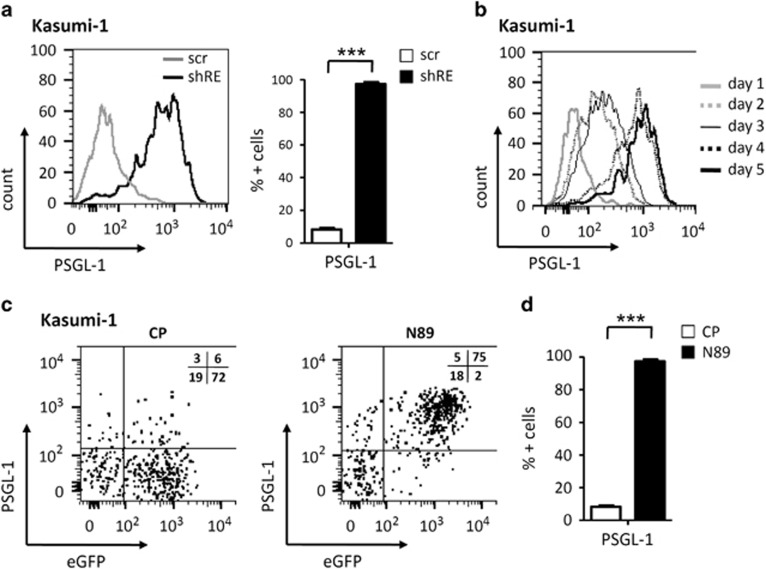
Effect of RE inhibition on PSGL-1 expression in Kasumi-1 cells. (**a**) Histogram and percentage of positive cells displaying cell surface PSGL-1 expression analyzed via flow cytometry in Kasumi-1 cells transduced with scramble control (scr) and shRNA against the breakpoint region of RE (shRE). (**b**) Cell surface levels of PSGL-1 expression over time after shRE transduction in Kasumi-1 cells. (**c**, **d**) Cell surface expression levels of PSGL-1 in Kasumi-1 cells transduced with lentiviral vectors expressing control peptides (CP), N89 peptides and eGFP as marker. Data shown as the mean±s.e.m. ***P<0.001. *n*=3.

To investigate whether inhibition of RE also affects PSGL-1 expression in a transformed RE-dependent human leukemic cell line, a short hairpin RNA (shRNA) against the breakpoint region of RE (shRE)^19^ was expressed in the RE-dependent cell line Kasumi-1. Compared with control cells expressing a mismatch control RNA (scr), RE depletion completely restored PSGL-1 levels in Kasumi-1 cells on day 4 post transduction, as observed in mobilized CD34+ cells (Figure 3a). PSGL-1 upregulation occurred within 24 h after lentivirus-mediated RE knockdown with increasing cell surface expression levels over time (Figure 3b). As tetramerization of RE is essential for its oncogenic function, we employed peptide-mediated interruption of RE tetramerization.^34, 36^ NLS-tagged NHR2 sequences were lentivirally overexpressed. NHR2 peptides are proposed to bind to the RE NHR2 domain, thereby blocking oligomerization of RE molecules and inhibiting its transcriptional and transforming properties.^34, 37^ Indeed, disruption of RE oligomerization using N89 peptides also activated PSGL-1 expression on the surface of Kasumi-1 cells to a similar extent (Figures 3c and d), thereby further supporting the notion that *PSGL-1* is a target gene of RE.

### RE interacts with and induces epigenetic modifications at the promoter region of *PSGL-1*

**Figure 4:**
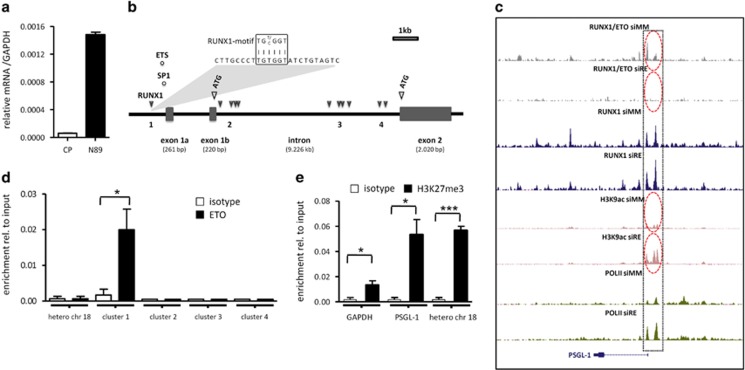
Transcriptional regulation of PSGL-1 expression in Kasumi-1 cells. (**a**) Expression levels of *PSGL-1* mRNA in Kasumi-1 cells transduced with control and N89 peptides as assessed via quantitative PCR. (**b**) *In silico* analysis shows RUNX1 recognition motifs in various regions of the *PSGL-1* gene sequences and the presence of SP1 and ETS transcription factor recognition motifs at the *PSGL-1* promoter region. (**b**, top) Alignment of the RUNX1 recognition motif (TG^T^/_c_ GGT) with the promoter sequences of the *PSGL-1* gene. (**c**) ChIP-sequencing data, which show RUNX1 and RE interaction with the promoter region of *PSGL-1*. SiRNA-mediated downregulation of RE facilitated RNA POLII-binding and H3K9ac marks at the upstream region of the *PSGL-1* gene. (**d**) Interaction of RE with the predicted RUNX1-binding clusters of the *PSGL-1* gene as analyzed via quantitative PCR, which amplified the ChIP DNA obtained against the RE-binding region. (**e**) H3K27me3 modification of the *PSGL-1* promoter region examined via ChIP analysis. The promoter region of *GAPDH* was used as negative control and the heterochromatin region of chromosome 18 (hetero chr 18) as positive control. Data shown as the mean±s.e.m. *P<0.05, ***P<0.001.

As lentivirus-mediated expression of RE-inhibiting peptides induced high mRNA levels of *PSGL-1* (Figure 4a), we next investigated the connection between RE binding and *PSGL-1* expression and examined *PSGL-1* genomic sequences for the presence of potential RUNX1-binding motifs (TGT/CGGT) *in silico.* Four clusters of RUNX1-binding motifs were identified at the promoter and within intronic regions (Figure 4b; Supplementary Figure 3a) together with Sp1 and ETS transcription factor-binding motifs within the predicted promoter region. ChIP-sequencing experiments using an ETO antibody^31^ confirmed several RE-binding sites as well as RUNX1 sites upstream of exon 1 of the *PSGL-1* genomic sequences, which disappeared after RE knockdown (Figure 4c). These data were verified by manual ChIP locating the RE-binding motif within the *PSGL-1* promoter region at −619 bp (cluster 1; Figure 4d; Supplementary Figures 2 and 3b). Among other adhesion molecules, PSGL-1 was identified also in human primary t(8;21)+ AML patient samples (see Supplementary Figure 4).^31, 35^ As RE has been shown to induce epigenetic changes to repress its target genes,^31^ we examined the RE-binding sites for the presence of several active and repressive histone marks including the repressive H3K27me3 chromatin mark. This revealed H3K27 trimethylation of the *PSGL-1* promoter upstream of exon 1 (Figure 4e). siRNA-mediated depletion of RE led to an increase in the binding of RUNX1 at the RE-binding sites with a concomitant increase in histone acetylation and RNA POLII within the predicted *PSGL-1* promoter region (Figure 4c; Supplementary Figure 3b). Overall, our data suggest that RE binding epigenetically represses PSGL-1 expression.

### RE-depleted Kasumi-1 cells gain potential to bind to P- and E-selectin under shear stress

**Figure 5:**
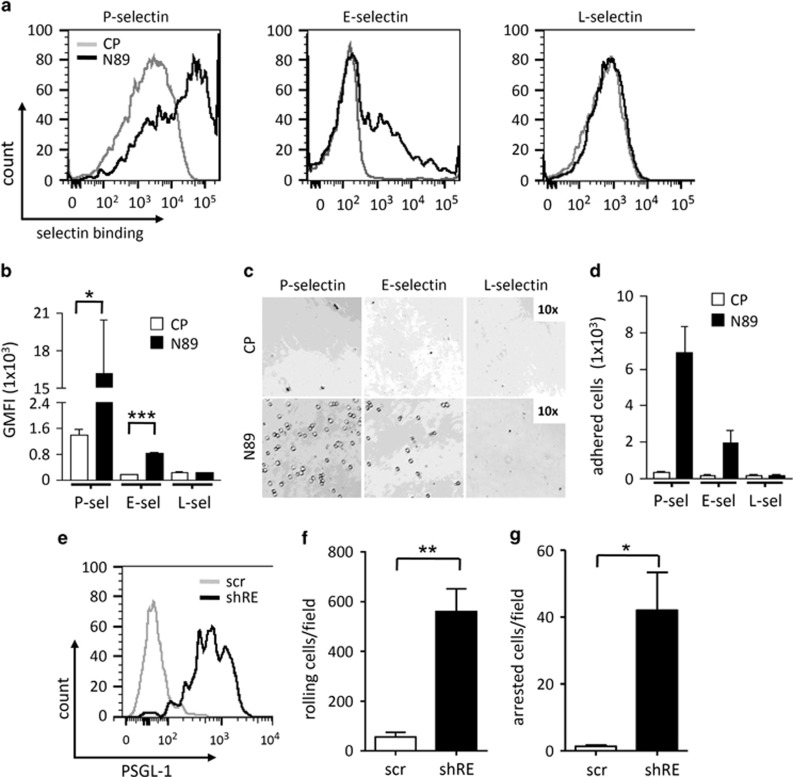
Functionality of restored PSGL-1 in shRE- and N89-treated Kasumi-1 cells. (**a**) Histograms of binding ability of Kasumi-1 cells transduced with CPs or N89 peptides to P-, E- and L-selectin as analyzed via flow cytometry and (**b**) percentage of corresponding positive cells. (**c**) Adhesion of CP- and N89-transduced Kasumi-1 cells on P-, E- and L-selectin-coated surfaces and (**d**) the corresponding quantitative values. (**e**) Cell surface expression levels of PSGL-1 in scramble (scr)- and shRE-transduced Kasumi-1 cells as analyzed via flow cytometry. (**f**) Rolling efficiency of shRE-transduced Kasumi-1 cells followed by (**g**) cell arrest on a P-selectin-coated surface under shear stress (2 dynes/cm^2^).

PSGL-1 is the principal ligand for P-selectin and, to a lesser extent, E-selectin^20^ but not L-selectin. We therefore investigated the consequences of RE inactivation for cell adhesion. The transduction of N89 inhibitor peptide led to a profound upregulation of binding of Kasumi-1 cells to P-selectin-coated beads and, to a lesser extent, E-selectin as measured via flow cytometry. However, no binding to L-selectin was observed (Figures 5a and b). Likewise, adhesion of N89-expressing Kasumi-1 cells was highly increased on P-selectin and, to a lesser extent, E-selectin-coated surfaces (Figures 5c and d). Furthermore, the capacity of shRE-expressing Kasumi-1 cells to interact with P-selectin-coated surfaces was assessed under shear stress. Before conducing the experiments, proper re-expression of PSGL-1 on the surface of shRE-transduced Kasumi-1 cells was confirmed (Figure 5e). Indeed, the rolling efficiency of shRE-treated Kasumi-1 cells on P-selectin-coated surface was also increased under shear stress at 2 dynes/cm^2^, whereas control cells showed only weak or no interaction (Figure 5f). Moreover, shRE-expressing Kasumi-1 cells were firmly arrested following rolling on P-selectin-coated surfaces (Figure 5g), thereby suggesting a strong interaction between the shRE-treated Kasumi-1 cells and P-selectin.

## Discussion

Our study adds important molecular details to previous studies demonstrating that the expression of RE perturbs the regulation of members of the adhesion gene family^17, 19^ by describing the consequences of RE binding for the regulation of *PSGL-1*, encoding for a mucin-like glycoprotein crucially involved in cellular adhesion. Together with the findings that in RE-expressing cells CD44 and VLA-4 are upregulated and CD11a is downregulated, this suggests that adhesion molecules are important targets for the establishment of a t(8;21)-specific cellular phenotype. A similar adhesion molecule pattern is found on highly mobile early hematopoietic myeloblasts in normal bone marrow. CD44 is broadly upregulated in AML and contributes to therapy relapse and has therefore been suggested as a potential therapeutic target.^17, 38^ CD11a is highly expressed on M4 and M5 leukemias, whereas M0 to M3 leukemias express the protein at low to intermediate levels.^39^ Similarly, PSGL-1 has been suggested as a marker to distinguish different types of AMLs.^40^ PSGL-1 is also involved in thymic settling of hematopoietic progenitors^41, 42^ as RE expression and *PSGL-*1 downregulation appear to be incompatible with T-cell progenitor homing to the thymus in spite of the presence of the fusion protein in myeloid cells and B cells from AML patients with t(8;21),^43^ indicating that the downregulation of this gene has dramatic consequences for cellular behavior, the most likely being an increased mobility of leukemic cells. Consistent with this idea, PSGL-1 is constantly downregulated in a large cohort of primary human AML M2 t(8;21)+ leukemia cells compared with bone marrow cells from healthy individuals. In an independent gene expression analysis, PSGL-1 was most prominently repressed in t(8;21)+ AML leukemias compared with various AML subgroups categorized by karyotypes, indicating that repression of this gene may be a core feature of core-binding factor leukemias. *PSGL-1* mRNA has indeed been found to be downregulated in t(12;21)+ AMLs expressing the fusion protein TEL/RUNX1, which has also been described as a dominant transcriptional repressor of RUNX1 target genes,^44^ suggesting that this fusion protein targets the same *cis*-regulatory elements.

To bind P- and E-selectin, PSGL-1 requires core 2 O-linked glycans that are sialylated and fucosylated. Interestingly, the sole derepression of PSGL-1 was fully sufficient for its adhesion function, thereby suggesting that PSGL-1-modifying components were activated in RE-depleted Kasumi-1 and hematopoietic progenitor cells. P- and E-selectin are responsible for adhesion of hematopoietic cells to endothelial cells.^45^ Interestingly, L-selectin, which is preferentially expressed on leukocytes, was not bound by upregulated PSGL-1 upon RE inhibition. These observations indicate that RE+ progenitors may have a reduced affinity to the vascular niche within the bone marrow.

Azab *et al.*^18^ have found increased PSGL-1 levels on malignant hematopoietic cells derived from multiple myeloma patients and showed that PSGL-1 has a critical role in the survival and development of multiple myeloma cells within the bone marrow.^46^ This stromal interaction was responsible for disease progression and drug resistance. Low-level PSGL-1 expression has an impact on the proliferation capacity of early hematopoietic stem cells as adhesion to P-selectin inhibits *in vitro* proliferation of human hematopoietic stem cells triggered by early acting growth factors.^47^ Furthermore, PSGL-1 has a role in stem cell anchorage within the bone marrow niche.^48^ RE-mediated repression of PSGL-1 may therefore have an impact on stem cell quiescence as well as leukemic engraftment. This is suggested by studies with PSGL-1-deficient BCR/ABL cells, which are impaired in engraftment potential in a mouse transplantation model.^49^ PSGL-1 deficiency also augments the mobilization of hematopoietic progenitor cells into the peripheral blood,^50^ thereby suggesting that RE-mediated PSGL-1 repression reduces cell adhesion in t(8;21)+ hematopoietic progenitor cells in the bone marrow. This may partially explain the favorable response of core-binding factor leukemias toward chemotherapy treatment.

Taken together, our data demonstrate a direct link between RE-binding and the pattern of expression of adhesion molecules in leukemic cells that will be of diagnostic relevance both as biomarker, but also for the evaluation of RE inhibitors that are currently under development.

## Materials and methods

### Cells, cell culture, viral production and viral transduction

FDCP-mix cells were cultured in IMDM medium (PAA Lab, Colbe, Germany) supplemented with horse serum (Gibco, Darmstadt, Germany) and murine IL3 (10 ng/ml; R&D Systems, Wiesbaden, Germany). Kasumi-1 cells were cultured in RPMI medium (Gibco) supplemented with 20% FCS (fetal calf serum; PAN Biotech, Aidenbach, Germany). Lin− mBM cells were cultured in StemSpan medium (StemCell Tech, Cologne, Germany) supplemented with murine IL3 (10 ng/ml), murine IL6 (50 ng/ml) and murine stem cell factor (50 ng/ml). Human CD34+ cells were cultured in StemSpan medium supplemented with human IL3 (10 ng/ml), human SCF (20 ng/ml), human IL6 (20 ng/ml), human FLT3L (20 ng/ml), human thrombopoietin (20 ng/ml) and human granulocyte–macrophage colony-stimulating factor (20 ng/ml). Lentiviral particles were produced using the calcium phosphate co-precipitation method. Briefly, 5.8 × 10^6^ HEK-293 T cells were seeded in a 10-cm^2^ tissue culture dish (Cellstar, Frickenhausen, Germany) and incubated at 37 °C in a humidified CO_2_ incubator. Next day, the co-transfection mix was prepared and distributed over the cells. Fresh medium was replenished 6 h after transfection. Finally, the viral supernatants were collected 48 h after transfection. Viral particles were then transduced into the cells on retronectin (50 μg/ml)-coated non-tissue culture plates.

### Retroviral vectors and shRNA against RE

Lentiviral LeGO vectors (http://www.lentigo-vectors.de/vectors.htm) co-expressing eGFP as a marker gene were described in our previous publication.^19^ Peptide-mediated interruption of RE tetramerization was performed using the NHR2-inhibitor peptide described before.^37^ Efficacy of the shRNA against the RE breakpoint sequences was recently demonstrated.^19^

### Isolation of human CD34+ and lin− mBM cells

Total murine bone marrow hematopoietic cells were harvested from 6- to 8-week-old Bl6 mice after killing via cervical dislocation under anesthetic conditions (isoflurane inhalation). The lin− mBM cells were enriched using the mouse lineage depletion kit (Miltenyi Biotec, Cologne, Germany). Mobilized human CD34+ bone marrow cells were obtained from healthy donors following their informed consent according to the institutional review board-approved protocol (DRK Blood donor service, Frankfurt am Main, Germany). Furthermore, the peripheral blood mononuclear cells were isolated via biocoll separation (Biochrom, Berlin, Germany). Thereafter, human CD34+ cells were enriched using a MACS CD34+ cell isolation kit (Miltenyi Biotec).

### Flow cytometry

To analyze expression of cell surface adhesion molecules, 1 × 10^5^ cells were prepared in 0.5% bovine serum albumin (100 μl) and incubated with FcR block (2 μl) for 10 min at room temperature. Thereafter, the cells were washed twice with 1x PBS. Subsequently, the cells were stained with fluorescent-labeled antibodies (1 μl antibody/100 μl cell suspension) and incubated for 30 min at 4 °C. Next, the cells were washed and measured via multicolor flow cytometry. The data were analyzed using FCS express and FlowJo software (Flowjo LLC, Ashland OR) under the institute's license.

### Quantitative PCR (qPCR)

*PSGL-1* expression at the mRNA level was analyzed via quantitative PCR. Briefly, 10^5^ −10^6^ cells were washed twice with ice-cold 1x PBS and centrifuged at 2000 r.p.m., at 4 °C for 5 min. The cell pellets were collected and processed for total RNA isolation using an RNasy kit (Qiagen, Hilden, Germany). Subsequently, the retrieved total RNA was treated with DNase I at 37 °C for 30 min. Thereafter, the enzymatic reaction was inactivated at 65 °C for 10 min. Furthermore, 500 ng of RNA was reverse-transcribed into cDNA for 1 h at 50 °C using oligo-dT primer and superscript III reverse transcriptase. Finally, 5% of the transcribed cDNA was used for gene expression analysis via qPCR using TaqMan reaction mixtures. The relative mRNA expression levels were calculated for each sample as follows: the mean of *PSGL-1* expression levels divided by the mean of *GAPDH* expression levels.

### Selectin-binding assay, cell rolling and arrest under shear stress

Recombinant P-, E- and L-selectin human chimeras (3 μg/ml) were conjugated with biotin (eBioscience, Frankfurt, Germany) followed by streptavidin (BD Pharmingen, Frankfurt, Germany). Transduced Kasumi-1 cells were incubated with the biotin/streptavidin-conjugated selectins and analyzed for selectin binding using a standard flow cytometry protocol. For cell adhesion, transduced Kasumi-1 cells were seeded on P-, E- and L-selectin (3 μg/ml)-coated non-tissue culture plates. After 2 h incubation at 37 °C, we gently washed the cells three times with 1x PBS. Then, adhered cells were quantified. The rolling and firm arrest efficiencies of transduced Kasumi-1 cells on its ligand P-selectin were assessed under shear stress. Briefly, the flow chamber slides were coated with P-selectin (5 μg/ml) for 30 min at room temperature. The P-selectin-coated surface was then incubated with 2% bovine serum albumin for 10 min. Next, 10^5^ cells were injected into the HBSS++ medium, which flows on the coated surface under the shear stress 2 dynes/cm^2^ applied simultaneously. Finally, the rolling and arrested cells on the P-selectin-coated surface were quantified.

### ChIP and ChIP-sequencing assay

DNA-binding ability of RE on the *PSGL-1* gene was assessed via ChIP assay. Briefly, 10^7^ Kasumi-1 cells were fixed. Protein/DNA complexes were crosslinked by adding 0.75% paraformaldehyde. Thereafter, the crosslinking reaction was terminated using 125 mM glycine. Subsequently, the cells were lysed using ChIP lysis buffer for 45 min at 4 °C after washing the cells in ice-cold 1x PBS. Meanwhile, 50 μl protein G beads were blocked with 100 × salmon sperm single-strand DNA (1%) in RIPA buffer at 4 °C for 30 min. After cell lysis, the cells were sonicated for 2 min to shear the chromatin to less than 1 kb in size. Next, 20–25 μg of sheared chromatin was incubated without the beads as an input control or with the pre-blocked protein G beads containing isotype (3 μg), ETO (5 μg), RNA polymerase II (2.5 μg) or H3K27me3 (2.5 μg) antibodies. After overnight incubation at 4 °C, the beads were collected under the magnetic field. The ChIP DNA was then eluted after reverse-crosslinking the chromatin using ChIP elution buffer. The RNA was degraded with RNase, and the protein was degraded with proteinase K. The DNA was isolated using a ChIP DNA isolation kit (Zymo research corp, Freiburg, Germany). Finally, the isolated DNA was amplified for *PSGL-1* gene sequences via qPCR. The *C*_t_ values were calculated for fold enrichment compared with the isotype control or input values. The forward (F) and reverse (R) primer sequences are as follows: cluster 1: ACCCTCACTTCTCTGGGTTCT (F), CACTCCATCCAGGTGTCACT (R); cluster 2: GCAACATGGTGAAACCTCGT (F), GAGTGCAGTGGCACAATCTC (R); cluster 3: ATAACTTGAGGCCAGGAGTTTG (F), CCGGGTTCAAGTGATTCTCC (R) and cluster 4: CCCAGACCACATCTCTGTGA (F), GGTACATGTGGCCCTTGC (R). The *GAPDH* and heterochromatin 18 primer sequences have been previously described.^31^ For the ChIP-seq experiments, anti-ETO antibody (Santa Cruz, sc-9737X, Santa Cruz Biotechnology, Wembley, UK) was used.^31^ The RUNX1 antibody (Abcam, ab23980, Cambridge, UK) recognizes the C-terminal domain of RUNX1, which is absent in the fusion protein. RE knockdown was archived using siRNA.^32^

### AML microarray set characteristics

Overall, 533 patients were treated within the AMLCG-99 trial (NCT00266136) and 29 patients within the M3-AMLCG study. 562 Microarrays: 140 Affymetrix HGU 133 2.0 plus and 422 A&B Set. A minimum of 20 metaphase cells were analyzed to consider the diagnosis of a normal karyotype (CN-AML). A complex karyotype was defined as the presence of three or more chromosome abnormalities in the absence of t(8;21), inv(16), t(16;16), t(15;17), t(9;11), t(v;11)(v;q23); inv(3), t(3;3) or t(6;9). Pretreatment bone marrow samples were prepared after Ficoll gradient centrifugation. Total RNA was extracted from 562 bone marrow samples as described previously^51^ and analyzed using Affymetrix HG-U133 A/B and 2.0 plus oligonucleotide microarrays (Affymetrix, Santa Clara, CA, USA). Hybridization and image acquisition followed official Affymetrix protocols. No cell sorting was performed. For probes to probe set annotation, we used custom chip definition files (CDFs) based on GeneAnnot version 2.0, synchronized with GeneCards Version 3.04 (available at http://www.xlab.unimo.it/GA_CDF/).^52^ These CDFs decrease the total number of probe sets (one probe set per gene), and potentially increase the specificity of the analyses by eliminating cross-hybridizing probes (probes are restricted by sequence specificity). Data normalization was performed using the Robust Multichip Average method as described before.^53^ Only the 17 389-probe sets present both on the A, B chips and the 2.0 plus chips were included in the analysis. Some probe sets on the A, B chips tend to have lower mean signal levels and higher s.d.'s than the corresponding probe sets on the Plus 2.0 chips. To eliminate this batch effect resulting from the different chip designs, we performed a second normalization using an empirical Bayesian method.
